# Mendelian randomization analysis reveals causal factors behind diabetic nephropathy: evidence, opportunities, and challenges

**DOI:** 10.3389/fendo.2024.1444808

**Published:** 2024-12-13

**Authors:** Qinchuan Huang, Chen An, Shiyun Tang, Yulin Leng, Yaowen Zhang, Bin Wan, Yutong Han, Yue Luo, Chunguang Xie

**Affiliations:** ^1^ Hospital of Chengdu University of Traditional Chinese Medicine, Chengdu, Sichuan, China; ^2^ Wangjing Hospital Affiliated to China Academy of Chinese Medical Sciences, Beijing, China; ^3^ Department of Endocrinology, Hospital of Chengdu University of Traditional Chinese Medicine, Chengdu, Sichuan, China; ^4^ Traditional Chinese Medicine (TCM) Regulating Metabolic Diseases Key Laboratory of Sichuan Province, Chengdu, Sichuan, China

**Keywords:** diabetic nephropathy, Mendelian randomization, bibliometrics analysis, genetic variation, causal relationship

## Abstract

Diabetic nephropathy (DN), as the most serious minor vascular complication of diabetes, imposes a significant socioeconomic and medical cost around the world, and its prevention and treatment are a major challenge in the current medical community. Observational studies and randomized controlled trials have revealed protective and risk factors for some DN. However, the conclusions of these researches may be influenced by several types of confounding. Mendelian randomization is a new epidemiological method mainly used to infer the causal relationship between exposure and outcome. Many Mendelian randomization studies have found potential causal relationships between DN and some diseases and lifestyle habits, thus providing valuable data for future mechanistic studies as well as the development and implementation of clinical prevention strategies. As a result, the purpose of this review is to evaluate the published Mendelian randomization study of DN, using the bibliometric research method, analyze the current research status and hot spots, and further summarize the genetic evidence about the potential protection of DN and risk factors to provide new inspiration for the etiology of DN and as a reference for clinical intervention.

## 1 Introduction

Due to ongoing changes in dietary habits and social structures, diabetes has become a major global health concern. Epidemiological surveys indicate that the number of people with diabetes worldwide will surpass 783 million by 2045 (1), and the prevalence rate has increased exponentially. Diabetic nephropathy (DN), also known as chronic kidney disease (CKD) caused by diabetes, is a microvascular complication frequently accompanied by massive proteinuria and retinopathy. Each year, more than one-third of newly diagnosed patients develop DN. DN accounts for 30% to 50% of end-stage renal disease cases, which is a leading cause of death and disability among diabetic patients (2).

Although the pathogenesis of DN is not clear, however, many randomized controlled trials (RCTs) and observational studies have found that genetic factors, intestinal flora, dietary lifestyle, and other factors are closely related to DN. However, promoting RCT testing is challenging due to discharge requirements and medical ethics constraints, and observational studies cannot eliminate many confounding factors, potentially leading to bias in the results (3). Consequently, no current studies can provide high-quality medical evidence to support preventive and treatment plans for DN.

The large sample Mendelian randomization (MR) research method, based on genome-wide association studies (GWAS), has gained widespread attention for studying high-risk factors of various diseases. To avoid the biased effects of confounding factors and reverse causality, MR analysis uses single nucleotide polymorphisms (SNPs) or genetic variations as instrumental variables (IVs) for causal inferences between risk factors and disease.

Using MR analysis to investigate high-risk factors for diabetes complications has become a research hotspot in recent years, driven by the public release of numerous large-scale GWAS studies. Among these, MR analysis related to DN is the most prominent and holds significant clinical value for understanding the etiology of DN. This review, therefore, focuses on the current situation of MR analysis in DN etiology to provide the basis and reference for the clinical prevention and treatment of DN.

## 2 An overview of the MR principle

Mendelian’s law of inheritance serves as the theoretical foundation for MR: during meiosis, genetic variation is assigned randomly to children and remains constant thereafter. MR analysis uses whole genome sequencing data and genetic variation as instrumental factors to determine the causal link between exposure and outcome.

**Figure 1:**
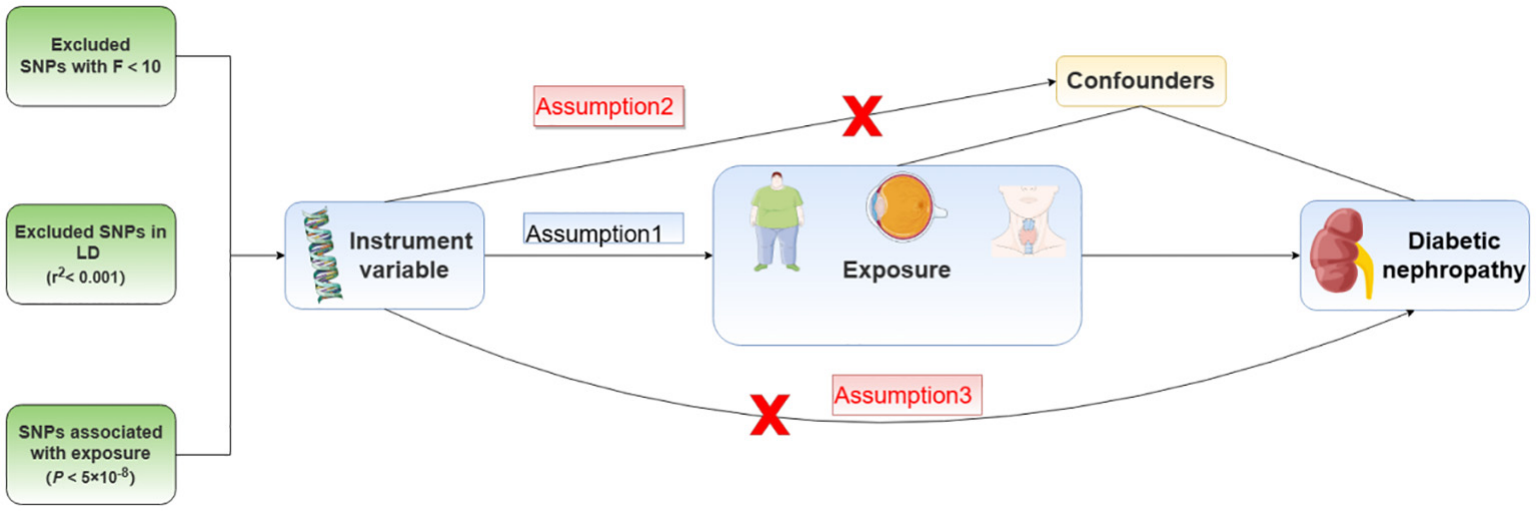
Mendelian randomization assumption model.

MR analysis requires that the selection of instrument variables should meet the three core assumptions of association, independence, and exclusivity: Assumption 1: instrument variables must be strongly associated with exposure factors; Assumption 2: instrument variables cannot be associated with any confounding factors associated with “exposure-outcome”; Assumption 3: instrument variables can only influence the outcome variables through exposure factors. Because genetic variation is independent of social environment, diet, and other factors, and the formation of genetic variation must precede the occurrence and change of various confounding factors and disease outcomes, using it as an instrumental variable can theoretically avoid the interference of confounding factors on the results while also avoiding the role of reverse causality (**Figure 1**).

MR analysis has gradually gained popularity in the medical field over the last decade, giving high-quality etiological evidence for a wide range of complicated clinical disorders as well as scientific guidance and reference for disease prevention and treatment.

## 3 Application and exploration of MR in the study of DN etiology

### 3.1 Bibliometric analysis of MR analysis related to DN

Bibliometric analysis can help to identify research trends and hotspots in existing fields, as well as inspire future studies.

This review was conducted by searching the Web of Science Core Collection (WOSCC). The search strategy is “((TS=(Diabetic kidney disease) OR TS=(Diabetic nephropathy)) AND TS=(Mendelian randomization)”, The search was finished by May 6, 2024, and after two scientists manually evaluated and removed papers unrelated to measuring risk factors for DN using MR analysis, a total of 90 articles matched the criteria. Based on R-Bibliometrix and Stork, the annual number of publications, published country map, and author cooperation network map were generated.

#### 3.1.1 Annual scientific publications

**Figure 2:**
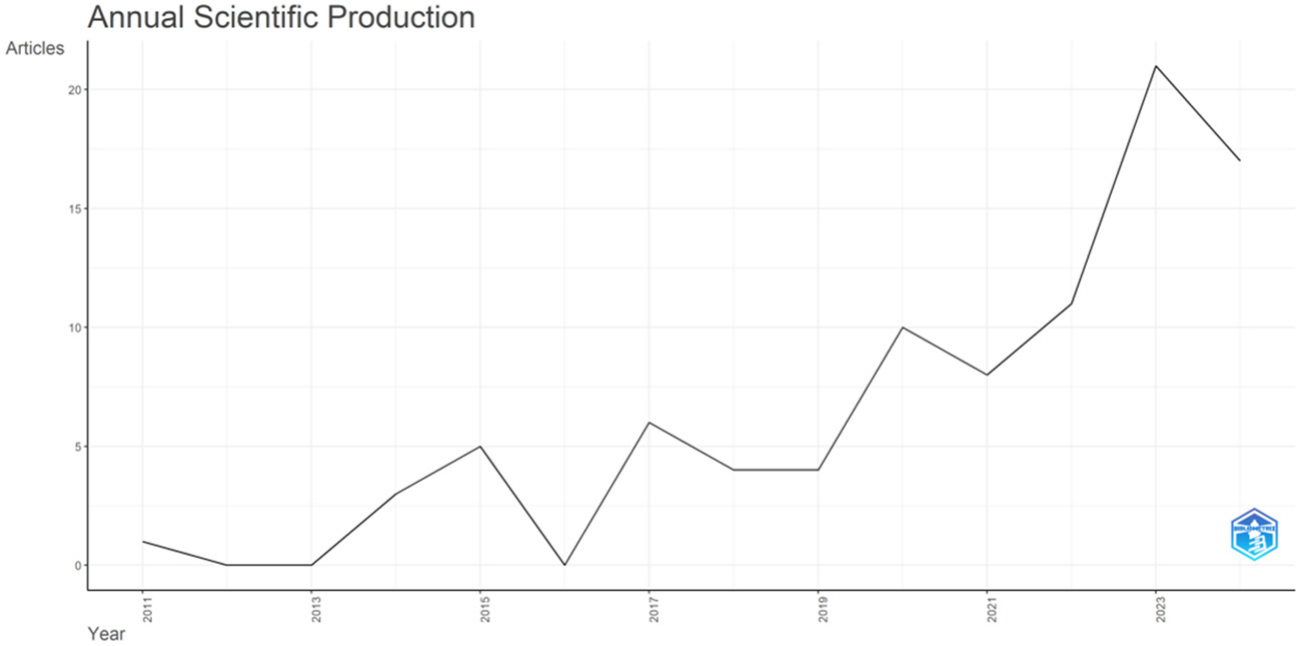
Annual scientific production of DN-related MR analysis.

Studies using MR analysis to explore the etiology of DN began in 2011. The number of publications has increased steadily over the last 13 years, with an overall yearly growth rate of 24.35%. From 2019 to 2024, the blowout stage of this field, the number of publications increased dramatically compared to previous years, peaking in 2023 (21 papers). Since the search was undertaken in May 2024, the 2024 publications count is incomplete; nonetheless, as of May, the publications count had reached 17 (**Figure 2**). It demonstrates that the use of MR analysis to investigate the high-risk variables of DN has gained widespread recognition.

#### 3.1.2 Country scientific production

**Figure 3:**
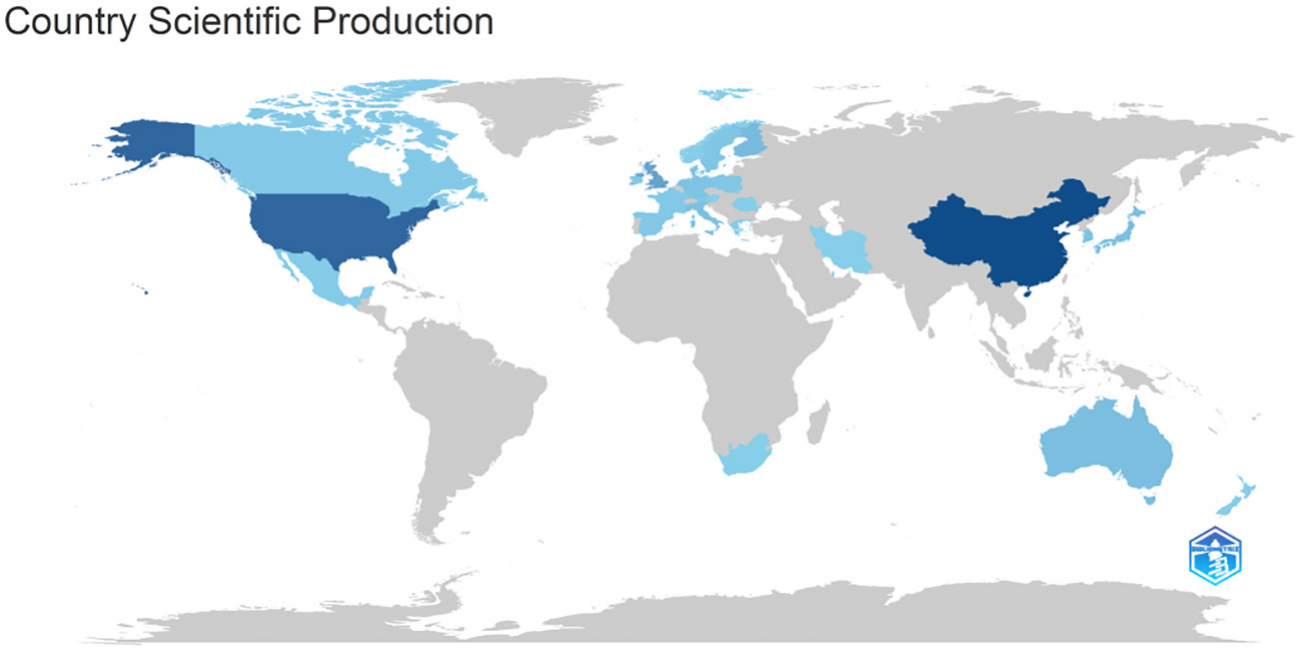
Country scientific production of DN-related MR analysis Darker colors indicate a higher number of productions; lighter colors indicate a lower number of production.

Using MR analysis to explore DN risk factors research primarily in North America, Europe, and Asia, including China’s largest post (44 papers), followed by the United States (13 papers) and Singapore (5 papers), according to the research map analysis, the field national research level difference is larger, with plenty of room for future development (**Figure 3**).

#### 3.1.3 WordCloud analysis

**Figure 4:**
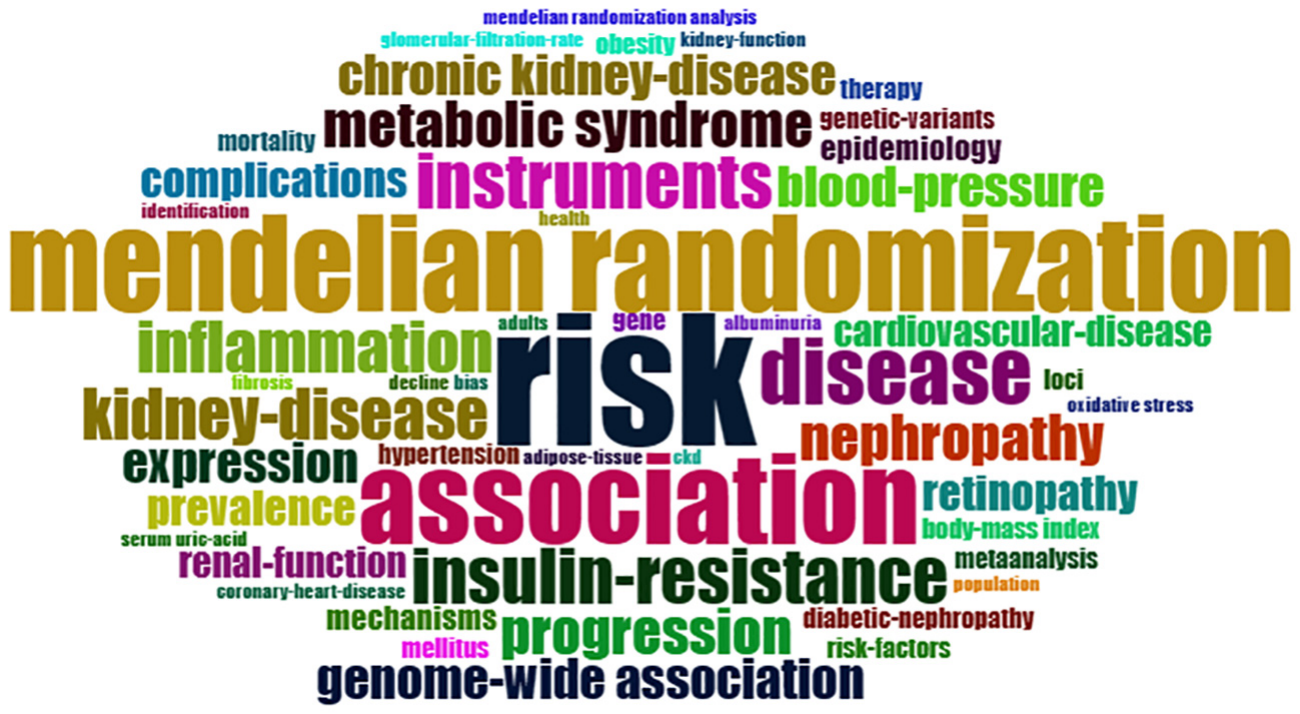
WordCloud of DN-related MR analysis.

Analyzing the keyword co-occurrence map can reveal the research hotspot in the current study topic. There is no doubt that “Mendelian randomization”, “risk” and “association” are the core keywords, but also include “inflammation”, “blood-pressure”, “insulin-resistance”, “cardiovascular-disease “ and other keywords (**Figure 4**), indicating that researchers have explored the causal relationship of various diseases and DN, trying to analyze the high-risk factors of DN progression provide a reliable basis and guidance for clinical preventive treatment.

#### 3.1.4 Researcher collaboration network

**Figure 5:**
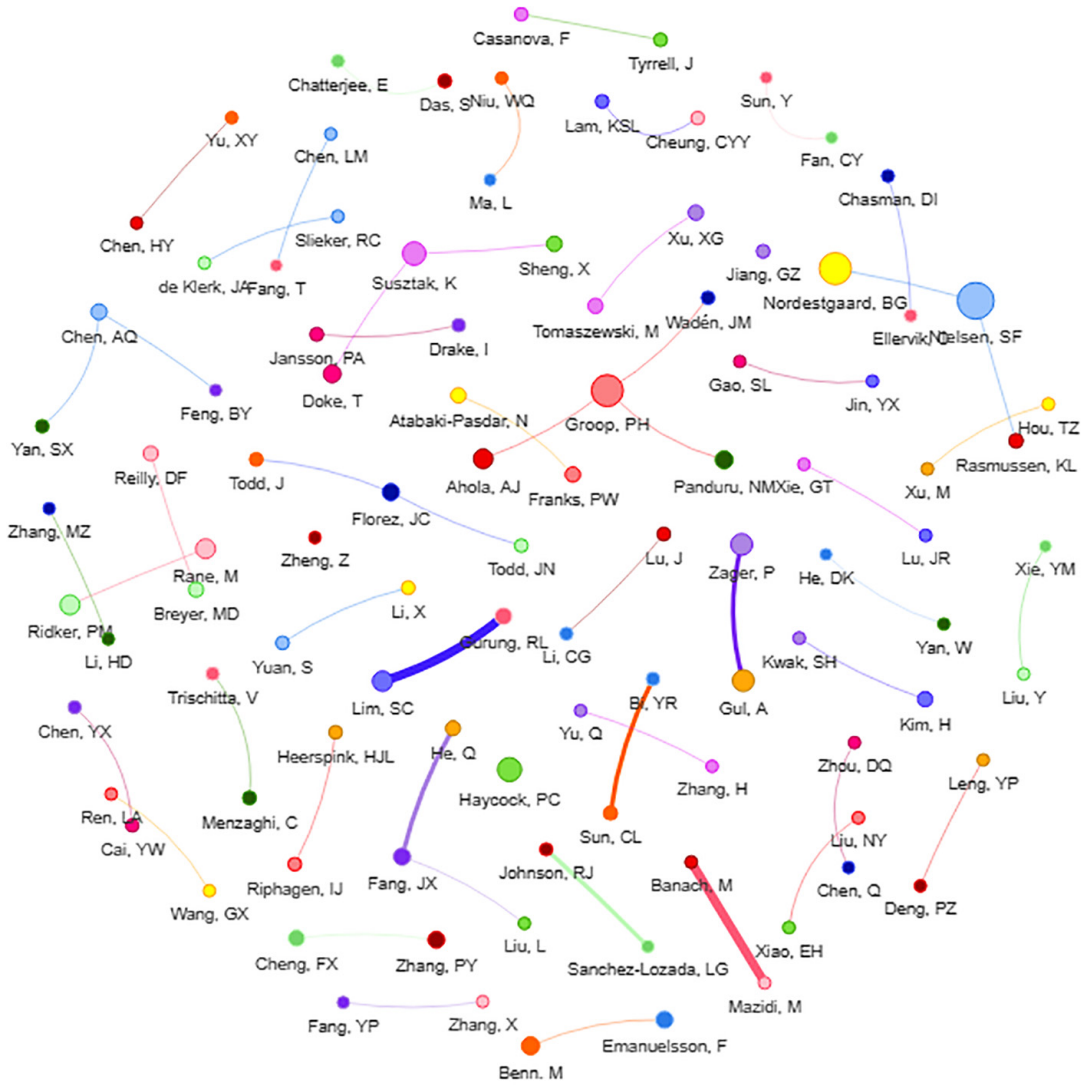
Researcher collaboration network of DN-related MR analysis Each dot represents a researcher, and the larger dot indicates higher output. The line between dots indicates that the researchers have cooperation, and the thicker line indicates the cooperation is closer.

Analyzing the researcher collaboration network can reveal the core authors in this subject, and knowing the important research of the core authors can aid in understanding the field ‘s future research trends. Liu is the most published author in this field (8 papers). Liu ‘s research mainly focuses on the study of micro-exposures such as leukocyte telomere length (4) and soluble receptors of advanced glycation end products (5), and their association with the risk of DN. The second was Groop (6 papers), who used the MR to analyze the risk of DN associated with various factors such as obesity (6) and serum uric acid concentration (7). Furthermore, this review discovered that researchers are closely cooperative, but only within their own region, and there is very little cooperation between researchers from various nations, implying that regional academic cooperation in this sector should be improved in future research processes (**Figure 5**).

#### 3.1.5 Publishing journal analysis

Analyzing publication journals offers a better understanding of key journals on the subject, which can be widely referenced in future research. The study finds that “Frontiers in Endocrinology” has the highest publication volume (15 papers), while “Kidney International” and “Diabetes Care” have the highest impact factor (IF = 14.8). Overall, journals publishing articles connected to Mendelian randomization analysis of DN display high standards, giving high-quality references for further related studies (**Table 1**).

**Table 1:** Main research journals of DN-related MR analysis (2023).

Journal	Count	Impact Factor	Journal Citation Reports
Frontiers in Endocrinology	15	3.9	Q2
Diabetes	7	6.2	Q1
Journal of Clinical Endocrinology & Metabolism	4	5.0	Q1
Diabetologia	3	8.4	Q1
Kidney International	2	14.8	Q1
Diabetes Care	2	14.8	Q1
Diabetes Metabolic Syndrome and Obesity	2	2.8	Q3
Frontiers in Microbiology	2	4	Q2
International Urology and Nephrology	2	1.8	Q3
BMJ Open Diabetes Research & Care	2	4.1	Q2

### 3.2 Causal relationship between micro-exposures and DN

#### 3.2.1 Gut microbiota

As a complex ecosystem, the intestinal microflora has a significant impact on the internal environment. The existence of the gut-kidney axis provides a possibility for the gut microbiota to contribute to the development of DN, and related studies have also found a close relationship between gut microbiota and DN (8–10). Therefore, disruptions in the gut microbiota are considered a risk factor for DN. However, various biases exist in observational studies, and the causal relationship remains unclear. Yan (11) obtained genetic data on gut microbiota from the publicly available GWAS data of the MiBioGen consortium, which includes 24 cohorts and 18,340 samples. Their study conducted an MR analysis on the relationship between 12 microbial taxa in gut microbiota and DN. The results showed that Akkermansia, Verrucomicrobia, Peptostreptococcaceae, Butyricimonas, Catenibacterium, and Marvinbryantia significantly increase the risk of DN. Among them, the lack of Akkermansia is closely related to obesity, diabetes, inflammation, and tumors (12). However, some studies have found that its abundance was positively correlated with serum creatinine (SCr) and blood urea nitrogen (BUN) levels (13). Therefore, it is speculated that Akkermansia may reduce the risk of developing diabetes by regulating glucose metabolism and intestinal function. However, with a prolonged duration of diabetes, Akkermansia may increase the risk of DN by affecting renal function. Verrucomicrobia and Akkermansia belong to the same bacterial group, and the mechanisms by which they increase the risk of developing DN may be similar. Therefore, further experiments are needed in the future to verify the role of gut microbiota at different stages of the disease. The above results were confirmed in the latest MR analysis by Yan (14).

#### 3.2.2 Blood biomarkers

As a complication of metabolic disease, observational studies have found that the progression of DN is closely associated with other metabolic markers in the body, including vitamin D, blood uric acid level, and serum albumin level, but the interference of confounding factors cannot be excluded. Therefore, conducting MR analysis can further elucidate the relationship between exposure and outcome, enabling better prediction of the progression of DN in the future.

Firstly, vitamin D participates in various signaling pathways within the body, including inflammation, apoptosis, proliferation, etc, in the form of 25-hydroxyvitamin D. Previous studies believe that vitamin D can improve glucose metabolism and inhibit the activation of the renin-angiotensin system (RAS) to achieve the purpose of preventing DN (15, 16). However, He et al. (17) conducted an MR analysis using SNP closely associated with vitamin D extracted from a publicly available GWAS involving 79,366 individuals of European descent. The study ultimately found no causal relationship between vitamin D and DN. Therefore, currently, from a genetic perspective, there is no support for using vitamin D supplementation as an effective strategy for preventing DN.

Secondly, uric acid, as the end product of purine metabolism, circulates through the kidneys and is excreted from the body, closely related to renal function. Epidemiological and observational studies have found an association between uric acid levels and the progression of DN (18, 19). However, in an MR analysis conducted by Ahola et al. (7) in 2014, contradictory results to previous studies were found. The study showed that there was no causal relationship between blood uric acid levels and the occurrence of DN in patients with type 1 diabetes (defined based on glomerular filtration rate) (OR: 2.24, 95% CI: -17.29 to 21.77, P = 0.631). Subsequently, Feng et al. (20) utilized the latest publicly available GWAS data from the FinnGen and UK Biobank to conduct an MR analysis, confirming these findings: there is no causal relationship between blood uric acid levels and DN. Our study speculated that uric acid may contribute to the progression of CKD by generating nitric oxide, activating the RAS, and stimulating vascular smooth muscle cell proliferation. However, for DN, simply lowering serum uric acid levels may not be beneficial for the outcome of DN.

Thirdly, serum albumin is the most abundant protein in the plasma. Previous cohort studies are ambiguous on the relationship between serum albumin and the onset of diabetes mellitus (21, 22). However, for DN, some relevant retrospective studies have found that the decreased serum albumin level is an independent risk indicator of DN (23, 24). Therefore, MR analysis is needed to avoid the possible error bias in previous studies to clarify the causal relationship. Cai et al. (25) combined prospective studies with bidirectional two-sample MR analysis. In the prospective study, it was found that for every 10 g/l increase in serum albumin levels, the hazard ratio (HR) of DN was 0.42 (95% CI: 0.30 to 0.58). Regarding the MR analysis, the researchers extracted 19 SNPs associated with serum albumin from the UK Biobank as instrumental variables. The MR analysis ultimately found a causal relationship between serum albumin levels and the occurrence of diabetes, which was negatively correlated (OR: 0.990, 95% CI: 0.984 to 0.995, P = 2.33×10^-4^), thus validating the results of the observational study. The protection mechanism of serum albumin for diabetes and DN may lie in its ability to regulate colloidal osmotic pressure and capillary membrane permeability. Additionally, it can scavenge free radicals and play an antioxidant role (26), In addition, the glycation of serum albumin may take precedence over the glycation of hemoglobin. Therefore, an increase in serum albumin levels can lead to enhanced competitive glycation inhibition, thus reducing the level of hemoglobin A1c (27). Ultimately, this contributes to lowering blood glucose levels and delaying the progression of microvascular complications.

#### 3.2.3 Inflammatory mediators

With the emergence of the inflammatory theory in the field of diabetic complications research, it has been receiving increasing attention. A growing body of experimental evidence emphasizes the significant role of inflammatory factors in the occurrence and progression of DN. Activated inflammatory cells migrate and infiltrate the kidneys, locally producing inflammatory mediators that exacerbate kidney damage in a high-glucose environment. Therefore, the elevation of inflammatory mediators may be an upstream event in the progression of DN. Clarifying the relationship between inflammatory mediators and DN is crucial for clinical prevention and treatment. An et al. (28) utilized two-sample MR to investigate the causal relationships between 41 inflammatory factors and DN. The final results revealed that Interferon-γ (IFN-γ) (OR: 1.33, 95% CI:1.09 to 1.63, P = 0.005) and Stem Cell Factor (SCF) (OR: 1.25, 95%CI: 1.02 to 1.52, P = 0.027) has a positive causal relationship with increased risk of DN, Meanwhile, Macrophage inflammatory protein-1β (MIP-1β) (OR: 0.92,95%CI: 0.85 to 0.98, P = 0.022) and interleukin-16 (IL-16) (OR: 0.89, 95%CI: 0.81 to 0.99, P = 0.043) were found to have a causal relationship with reduced risk of DN. Furthermore, Lin et al. (29) conducted an observational study and used genetic data provided by the Taiwan Biobank for MR analysis. The final results of both studies indicate a causal relationship between levels of high-sensitivity C-reactive protein (hs-CRP) and the occurrence of DN.

This review analyzed the roles of relevant inflammatory mediators in DN as follows: First, IFN-γ overexpression increases IFN-regulatory factors (IRFs), as well as the secretion of nuclear factor kappa-B (NF-κB) and signal transducer and activator of transcription-1 (STAT-1), selectively promoting the polarization of M1 macrophages (30). This leads to increased secretion of inflammatory cytokines. Furthermore, IFN-γ can also enhance the expression of vascular endothelial growth factor (VEGF). Through the combined action of these mechanisms, continuous damage to the renal microvasculature may occur. Observational studies further confirmed that elevated IFN- γ is a predictor of DN onset (31). For SCF, the SCF/c-kit signaling pathway leads to the aggregation of endothelial progenitor cells and promotes angiogenesis. Animal experiments have found a significant positive correlation between the expression levels of SCF and c-kit and the degree of mast cell infiltration. Mast cell infiltration promotes renal interstitial fibrosis, thereby increasing the risk of DN (32). Finally, CRP is a nonspecific marker of inflammation and tissue damage. CRP can promote renal inflammation through the CD32b-NF-κB signaling pathway and induce renal fibrosis via the CD32b-Smad3-mTOR signaling pathway (33). Additionally, elevated CRP levels increase the production of pro-inflammatory cytokines, leading to mesangial cell proliferation, excessive matrix production, and increased vascular permeability, which in turn results in renal function impairment and albuminuria (34). The aforementioned MR results emphasize that early intervention targeting certain inflammatory mediators can play a positive role in protecting patients with DN.

#### 3.2.4 Leukocyte telomere length

Telomeres are DNA-protein structures at the ends of chromosomes, and their length shortens with each cell division. Telomeres protect the genome from damage and serve as important markers of organismal aging and cellular apoptosis. A study has found that controlling telomere length is crucial for maintaining telomere stability (35). A cohort observational study found that relatively short telomere length (RTL) is closely associated with faster CKD progression in diabetic patients (HR: 1.16, 95% CI: 1.01 to 1.34, P = 0.03) (36). Another prospective study based on multiple Asian ethnicities similarly found that T2DM patients with shorter leukocyte telomere length (LTL) had more than double the risk of albuminuria (OR: 2.10, 95% CI: 1.15 to 3.83, P = 0.016) (37). These studies suggest that short LTL may be a novel biomarker for the progression of DN. To further clarify the causal relationship, Gurung et al. (4) extracted 16 SNPs closely associated with leukocyte telomere length (LTL) from a GWAS in the Singapore Chinese Health Study (SCHS) cohort and conducted a two-sample MR analysis. The final results validated the conclusions of previous observational studies: genetically determined shorter LTL is closely associated with an increased risk of CKD in T2DM patients (OR: 1.51, 95% CI: 1.12 to 2.12, P = 0.007). Therefore, preventing premature telomere shortening will be an important strategy in the prevention and treatment of DN.

#### 3.2.5 Drug target

Drug-target MR analysis is emerging as an effective tool for inferring the effect of various drugs acting on encoded proteins on disease risk (38). Unlike traditional MR studies, in drug-target MR analysis, genetic variants are selected from the gene of interest or a neighboring genomic region. The wide application of drug-target MR can help to better identify the potential targets of drug action on diseases in order to facilitate drug development and improve clinical efficacy.

For the onset and progression of DN, the renin-angiotensin-aldosterone system (RAAS) plays an important role, and inhibition of the RAAS can have many positive effects on the prevention of DN (39). RAAS inhibitors, with Angiotensin Converting Enzyme Inhibitors (ACEI) and Angiotensin Receptor Blockers (ARB), are the most widely used in clinical practice. However, a follow-up study found that DN patients who have been treated with ARB, are still at high risk of end-stage renal disease (ESRD) outcomes (40). In addition to this, another study found that combining an ACEI with an ARB increased the risk of hyperkalemia and acute kidney injury (41). Thus, drug-target MR offers the possibility to explore the target mechanism of action of RAAS inhibitors in DN. Zhou (42) conducted a network pharmacology combined with a drug-target MR study, and the final MR results demonstrated the positive effects of CTSC (IVW, OR: 0.861, P=0.041) and PDE5A (IVW, OR: 0.842, P=0.018), the key targets of RAAS inhibitors in the treatment of DN, in protecting DN. CTSC has been shown in previous studies to be a human DN uroprotein gene, which may play a role by participating in the regulation of urinary proteins (43). PDE5A belongs to the phosphodiesterase family, and an animal study has shown that targeted regulation of PDE5 exerts a significant anti-fibrotic and nephroprotective effect on the kidney (44).

In addition, statins are widely used in the diabetic population because diabetic patients are often prone to comorbid lipid metabolism abnormalities (45). However, previous studies have differed on the use of statins in patients with DM. A multicenter cohort study conducted in China showed that statin use was associated with a lower risk of DN events (HR: 0.72, 95% CI = 0.62 to 0.83) (46). A retrospective cohort study, however, found that statin use in diabetic patients was associated with a moderately elevated risk of kidney disease progression (OR: 1.16, 95% CI:1.12 to 1.20) (47). Therefore, drug-target MR studies need to be introduced to avoid the influence of various confounding factors on the results. Zhao et al. (48) found a significant correlation between HMGCR inhibition, the main pathway of action of statins, and a high risk of DN (OR: 1.79, 95% CI:1.14 to 2.78, P = 0.01). The results of MR are usually interpreted as lifetime exposure, so the final results can be interpreted as a negative effect of long-term HMGCR inhibitors on DN. This study provides new insights into the selection of lipid-lowering medications for patients with clinical DN.

#### 3.2.6 Proteomic

Proteomic MR is an emerging research direction with similarities to drug-target MR, as proteins often have specific binding sites or regions that can be targeted by biologics, so with the identification of thousands of protein quantitative trait loci (pQTLs) for plasma proteins by GWAS, carrying out proteomic MR can help us to identify the potential therapeutic sites for DN and identify drug targets in advance. Fan et al. (49) conducted a proteomic MR by extracting pQTL of plasma proteins from seven different proteomic GWAS and found that higher levels of MICB (OR: 1.46, 95% CI 1.27 to 1.67; P = 3.94×10^-8^), GZMA (OR: 1.34, 95% CI 1.17 to 1.53; P = 1.86×10^-5^), and CLIC5 (OR: 1.45, 95% CI 1.04 to 2.03, P = 2.99×10^-2^) may promote the progression of DN, whereas CTSS (OR: 0.90, 95% CI 0.83 to 0.97, P = 5.78×10^-3^) may play a protective role in the progression of DN. Zhang et al. (50) subsequently combined proteomic MR with co-localization analysis and external validation in an attempt to find key targets for the treatment of DN, resulting in the novel identification of potential drug target properties of COL6A2, CBLN1, TGFBI, and ITIH3 for the treatment of DN. Gurung et al. (51) conducted an MR analysis based on young Asian T2DM DN patients and found that higher plasma ANG levels were associated with an increased risk of DN in young Asian T2DM (OR: 4.03, 95% CI 1.28 to 12.68, P = 0.017).

These proteomic MR studies offer the possibility of new protein targets for the prevention and treatment of DN.

### 3.3 Causal relationship between macro-exposures and DN

#### 3.3.1 The underlying diseases

The occurrence and progression of DN are regulated and influenced by the endocrine system, immune system, and other factors. Using MR analysis to study the causal relationship between various diseases and DN contributes to the early diagnosis and screening of DN in populations with related diseases, enabling early and effective intervention.

Firstly, Diabetic Retinopathy (DR) is another typical microvascular complication of diabetes mellitus, apart from DN. Meta-analysis has shown that the comprehensive sensitivity and specificity of diabetic retinopathy in predicting DN are 0.65 (95% CI: 0.62 to 0.68) and 0.75 (95% CI: 0.73 to 0.78), respectively. Duan et al. (52) further conducted MR analysis by extracting SNPs closely related to DR and DN from FinnGen and UK Biobank. They ultimately found a causal relationship between DR and DN occurrence (OR: 2.89, 95% CI: 1.76 to 4.75, P<0.001), validating the conclusions of previous observational study (53). Therefore, it is recommended to promptly perform comprehensive renal function tests in DR patients to prevent the occurrence of DN.

Thyroid hormone receptors are abundantly present in the vascular endothelium, and fluctuations in thyroid hormone levels can affect vascular function. Although current observational studies have found a certain correlation between thyroid function and renal function (54, 55), there is still insufficient evidence to prove a direct causal relationship between thyroid function and DN. Therefore, Li et al. (56) conducted an MR analysis based on a European population sample to investigate the causal relationship between thyroid function and DN. They found that thyroid-stimulating hormone (TSH) was positively correlated with the risk of DN (OR: 1.44, 95% CI: 1.04 to 2.41, P = 0.033). Additionally, TSH was negatively correlated with the estimated glomerular filtration rate (eGFR) in diabetic patients (β: -0.031, 95% CI: -0.063 to -0.001, P = 0.047). This suggests that an increase in TSH may raise the risk of DN and simultaneously decrease the eGFR in patients with T2DM. This study speculates that this is because TSH can stimulate leptin secretion, thereby increasing hepatic glucose output and enhancing gluconeogenesis to stimulate endogenous glucose production, which in turn reduces hepatic insulin sensitivity. Additionally, TSH inhibits insulin synthesis in β-cells, ultimately raising blood glucose levels (57). Moreover, abnormal secretion of thyroid hormones combined with a high-glucose environment will further exacerbate damage to the vascular endothelium (58). Although MR analysis has demonstrated the causal relationship between them, further basic research is still needed to clarify the underlying mechanisms in the future.

Obesity has been considered a starting point for multiple diseases and is closely associated with the occurrence of complications in diabetes. Assessment of obesity includes indicators such as body mass index (BMI), waist circumference (WC), trunk fat content, etc. Several MR analyses have now demonstrated a significant causal relationship between obesity and DN (6, 59–61). Our study suggests that obesity leads to reduced secretion of adiponectin in the body. Adiponectin has various beneficial effects, such as anti-atherosclerosis and anti-inflammatory properties (62). Consequently, reduced adiponectin levels exacerbate endothelial damage and inflammation. Additionally, excessive visceral adipose tissue (VAT) can disrupt metabolism and exacerbate insulin resistance. Meanwhile, lymphocytes and macrophages infiltrate adipose tissue, leading to the release of inflammatory cytokines and reactive oxygen species, exacerbating the body’s inflammatory response and oxidative stress levels, and ultimately promoting the progression of DN (63, 64).

Additionally, after MR analysis, diseases such as inflammatory bowel disease (65), sarcopenia (66), and periodontitis (67) were found to lack a significant causal relationship with DN from a biogenetic perspective.

#### 3.3.2 Lifestyle

Since the 21st century, there has been a significant change in human lifestyle compared to previous times. Factors such as diet and daily routines constantly influence bodily functions, leading to the emergence of a new discipline: Lifestyle Medicine. This discipline aims to study the significance of lifestyle changes in the prevention and treatment of chronic diseases (68). Using MR analysis to explore the relationship between lifestyles and DN effectively avoids errors caused by confounding, reverse causation, and bias. It has become an essential tool in epidemiological research today.

Coffee, currently the most widely consumed beverage worldwide, contains main components such as caffeine, chlorogenic acid, and hydroxy-hydroquinone (69). Previous epidemiological studies and meta-analyses have not reached consensus on the relationship between coffee consumption and diabetes and its complications. Some studies suggest that coffee can significantly reduce the risk of developing T2DM while delaying the occurrence of diabetic complications (70–72). However, a prospective study has found that consuming more than 2 cups of caffeine-containing beverages per day increases the risk of eGFR decline by 1.19 times (OR: 1.19, 95% CI: 1.01 to 1.41) (73). Therefore, further research is needed to clarify the relationship. In 2021, Mazidi et al. (74) conducted an MR analysis on the relationship between coffee intake and kidney function, finding no causal relationship between coffee intake and eGFR in diabetic patients. Additionally, in 2023, an MR analysis based on the latest GWAS data from the UK Biobank revealed a causal relationship between coffee consumption and the risk of DN, showing a positive correlation (OR: 1.939, 95% CI: 1.012 to 3.712, P = 0.045) (75). Therefore, based on the results of MR analysis and genetic perspective, it is suggested that coffee does not provide a protective effect against the occurrence of DN. Moreover, excessive intake of coffee may increase the risk of DN. This review suggests that this is because caffeine, as an adenosine receptor antagonist, binds to adenosine receptors upon intake (76), influencing adenosine’s anti-inflammatory properties and glomerular hemodynamics, leading to glomerular remodeling, sclerosis, and the occurrence of proteinuria (77).

Sleep plays a crucial role in the regulation of endocrine functions (78). Previous observational studies have found significant associations between both long and short sleep durations and the occurrence of DN, as well as increased levels of urinary albumin-to-creatinine ratio (UACR) (79, 80). To further clarify the role of sleep in DN, Mazidi et al. (81) selected 78 SNPs closely related to sleep duration from the Biobank as instrumental variables for MR analysis. The final results, however, revealed no causal relationship between sleep duration and eGFR levels in T2DM patients. However, the study also found that, in non-diabetic populations, longer sleep durations were causally associated with lower eGFR levels (IVW: β: -0.024, SE = 0.011, P = 0.020). This suggests that prolonged sleep may have potential adverse effects on renal function. Our analysis suggests that sleep duration is closely related to the levels of various inflammatory factors (tumor necrosis factor-alpha, IL-1, CRP, etc.) (82). At the same time, disruption of sleep rhythms can negatively impact the RAS, sodium-potassium excretion system, and thereby damage renal function (83). A study in animal models has found that circadian disruption (including prolonged, shortened, or interrupted sleep) leads to proteinuria, glomerulosclerosis, tubular hyperplasia, and renal fibrosis (84).

## 4 Limitations and prospects of using MR in DN etiology research

Although MR analysis has been widely applied in the field of DN etiology research, providing definite advantages for exploring DN risk factors, there are still certain limitations and challenges in its future application. The summary is as follows: First of all, MR is dependent on the gene-exposure-disease chain, and if the effect of one link is weak, the effectiveness of the overall analysis will be affected. GWAS is a genome-wide association study of genes and phenotypes, and therefore, large samples and high representativeness of GWAS data are required for the genetic data to have authenticity and persuasive power. However, the number of cases of GWAS data is relatively small, resulting in fewer instrumental variables to be included in the study, which will reduce the effectiveness and specificity of the analysis. Furthermore, most of the current GWAS data are derived from UK Biobank, whose study population is predominantly of European origin, which may further limit the extrapolation of the results. Therefore, the generalizability of the results and the impact of possible sample overlap on the results need to be considered when conducting MR. In future studies, GWAS data with larger sample sizes should be preferred for analyses, and the sample sizes of different populations and cohorts with relevant exposures and DN should also be continuously expanded to guarantee the authenticity and reliability of the study. Secondly, due to the inconsistency of diagnostic criteria for DN, the gold standard for pathology is still needed to assess whether CKD is due to diabetes. Therefore, further improvement of DN phenotype information as well as data on its genetic variants is needed in the future. Finally, although MR can reveal the causal relationship between exposure and outcome, it is still unable to elaborate and analyze the specific mechanism of action, so in the future, it is still necessary to combine with basic studies, RCT, etc. to explore the specific pathways and targets of exposure so as to better guide the prevention and control of disease and to provide high-quality evidence-based evidence for the diagnosis and treatment of disease in the later stage.

## 5 Conclusion

**Figure 6:**
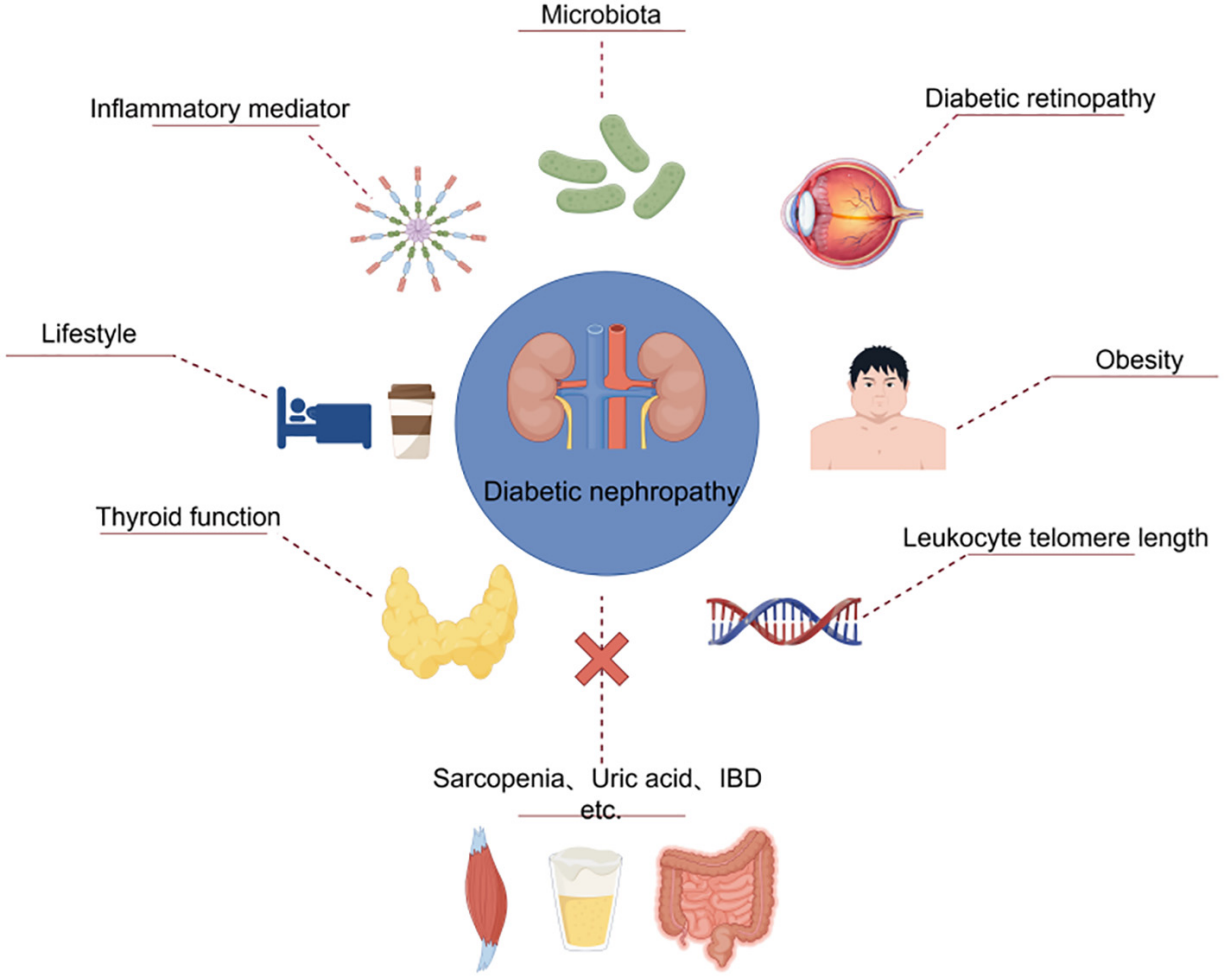
Risk factors for DN in the MR analysis.

The article provides a review of recent studies on the application of MR analysis in DN epidemiology research, summarizing the causal relationships between various exposure factors and the risk of DN. Ultimately, this review found that gut flora such as Akkermansia and Verrucomicrobia, serum albumin levels, inflammatory mediators such as IFN-γ and CRP, leukocyte telomere length, protein, diabetic retinopathy, thyroid dysfunction, obesity, coffee intake, and sleep were all causally associated with the development of DN (**Figure 6**). However, based on current genetic data, MR analysis failed to prove that vitamin D, uric acid, inflammatory bowel disease, sarcopenia, periodontitis, etc. are risk factors for DN (**Table 2**). MR analysis plays a groundbreaking role in enhancing researchers’ understanding of DN etiology and developing new treatment approaches. Based on this, early intervention and prevention of relevant risk factors in clinical diagnosis and treatment processes can be conducted. The ultimate goal is to help prevent DN in high-risk populations while slowing the progression of the disease in people with DN.

**Table 2:** Mendelian randomization studies of various exposures with DN as the outcome.

Exposures	Year	Authors	Population of exposure	Population of outcome	SNPs, n	Effect	P-value
Verrucomicrobia	2023	Yan W et al. (11)	18,340 adult individuals, European	DN, 210463 controls and 3283 cases, European	12	OR = 1.390(1.10–1.75);	P= 0.005
Akkermansia	2023	Yan W et al. (11)	18,340 adult individuals, European	DN, 210463 controls and 3283 cases, European	12	OR=1.390(1.10–1.75)	P= 0.005
Peptreptococcaceae	2023	Yan W et al. (11)	18,340 adult individuals, European	DN, 210463 controls and 3283 cases, European	14	OR=1.284(1.03–1.59)	P= 0.012
Butyricimonas	2023	Yan W et al. (11)	18,340 adult individuals, European	DN, 210463 controls and 3283 cases, European	16	OR = 1.261( 1.02–1.55)	P= 0.031
Catenibacterium	2023	Yan W et al. (11)	18,340 adult individuals, European	DN, 210463 controls and 3283 cases, European	4	OR=1.278(1.02-1.59)	p=0.030
Marvinbryantia	2023	Yan W et al. (11)	18,340 adult individuals, European	DN, 210463 controls and 3283 cases, European	10	OR = 1.369(1.04–1.79)	P= 0.022
Class Verrucomicrobiae	2024	Yan S. et al. (14)	18,340 adult individuals, European	T2DN, 283224 controls and 2394 cases, European	9	OR=1.822 (1.241-2.676)	P=0.013
Eubacterium protogenes	2024	Yan S. et al. (14)	18,340 adult individuals, European	T1DN, 283224 controls and 1441 cases, European	13	OR=0.407(0.241-0.688)	P=0.002
25(OH) vitamin D	2023	He M. et al. (17)	79,366 adult individuals, European	T1DN (early), 67452 controls and 3399 cases, European	3	OR=0.587(0.03 -11.458)	P=0.726
25(OH) vitamin D	2023	He M. et al. (17)	79,366 adult individuals, European	T1DN (later), 67452 controls and 4352 cases, European	3	OR=1.517(0.114-20.208)	P=0.752
25(OH) vitamin D	2023	He M. et al. (17)	79,366 adult individuals, European	T2DN (early),2238 controls and 1989 cases, European	3	OR=0.039(0.114-20.208)	P=0.109
25(OH) vitamin D	2023	He M. et al. (17)	79,366 adult individuals, European	T2DN (later), 2372 controls and 1339 cases, European	3	OR=1.870(0.389-8.990)	P=0.435
Serum uric acid	2017	Ahola AJ. et al. (7)	>140,000 adult individuals ,European	T1DN (eGFR), 2720 cases, European	23	OR=2.24 (-17.29-21.77)	P= 0.631
Serum uric acid	2022	Feng B. et al. (20)	336619 adult individuals, European	DN, 210463 controls and 3282 cases, European	188	OR=1.06(0.91-1.24)	P=0.428
Serum Albumin	2023	Cai YW. et al. (25)	115,060 adult individuals, European	T2DM, 439,238 controls and 22,340 cases, European	19	OR=0.990 (0.984-0.995)	P=2.33×10-4
IFN-γ	2024	An L. et al. (28)	8293 adult individuals, European	DN, 210463 controls and 3282 cases, European	13	OR=1.33(1.09-1.63)	P=0.005
SCF	2024	An L. et al. (28)	8293 adult individuals, European	DN, 210463 controls and 3282 cases, European	9	OR=1.25(1.02-1.52)	P=0.027
MIP-1β	2024	An L. et al. (28)	8293 adult individuals, European	DN, 210463 controls and 3282 cases, European	17	OR=0.92(0.85-0.98)	P=0.022
IL-16	2024	An L. et al. (28)	8293 adult individuals, European	DN, 210463 controls and 3282 cases, European	10	OR=0.89(0.81-0.99)	P=0.043
hs-CRP	2023	Lin CC. et al. (29)	2332 adult individuals, Chinese	DN, 2332 controls and 256 cases,chinese	4	OR=1.67(1.40-1.98)	NOT REPORT
Leukocyte telomere length	2021	Gurung RL. et al. (4)	25,273 East Asians and 37,505 European	T2DN, 2005 controls and 498 cases,East Asians	16	OR=1.51(1.12-2.12)	P=0.007
Diabetic retinopathy	2023	Duan J. et al. (52)	95752 adult individuals, European	DN, 210463 controls and 3283 cases, European	4	OR=2.89(1.76-4.75)	P<0.001
TSH	2023	Li H. et al. (56)	39282 adult individuals, European	DKD, 3,676 cases and 283,456 controls, European	39	OR=1.44(1.04-2.41)	P=0.033
FT4	2023	Li H. et al. (56)	72,167 adult individuals, European	DKD, 3,676 cases and 283,456 controls, European	16	OR=0.830.67-1.03)	P = 0.093
TPOAb	2023	Li H. et al. (56)	>40000 adult individuals, European	DKD, 3,676 cases and 283,456 controls, European	4	OR=1.17 (0.57-2.38)	P = 0.672
Obesity	2015	Todd JN. et al. (6)	249796 adult individuals, European	T1DN macroalbuminuria, 2347 cases and 6049 controls, European	NOT REPORT	OR 1.28(1.11-1.45)	P = 0.001
Obesity	2015	Todd JN. et al. (6)	249796 adult individuals, European	T1DN ESDR,2347 cases and 6049 controls, European	NOT REPORT	OR 1.43(1.20-1.72)	P < 0.001
Obesity	2015	Todd JN. et al. (6)	249796 adult individuals, European	T1DKD ,2347 cases and 6049 controls, European	NOT REPORT	OR 1.33(1.17-1.51)	P < 0.001
Body Mass Index	2023	Huang Y. et al. (59)	461460 adult individuals, European	DN, 3283 cases and 210463 controls, European	376	OR=1.74(1.47-2.07)	P=0.000000000217
Waist circumference	2023	Huang Y. et al. (59)	462166 adult individuals, European	DN, 3283 cases and 210463 controls, European	315	OR=2.03(1.62-2.55)	P=0.0000000011
Body Mass Index	2022	Wang M. et al. (60)	681275 adult individuals, European	DN, 3,283 ncase 181,704 controls, European	441	OR=1.99 (1.47–2.69)	p = 7.89 × 10−6
Waist circumference	2022	Wang M. et al. (60)	232101 adult individuals, European	DN, 3,283 ncase 181,704 controls, European	214	OR=2.48 (1.40–4.42)	p = 1.93 × 10−3
Trunk fat mass	2022	Wang M. et al. (60)	454588 adult individuals, European	DN, 3,283 ncase 181,704 controls, European	34	OR=1.80 (1.28–2.53)	p = 6.84 × 10−4
Body Mass Index	2022	Lu J. et al. (61)	158284 adult individuals,Japanese	DN,1314 cases and 2658 controls,chinese	56	OR=3.76(1.88-7.53)	P < 0.001
Inflammatory bowel disease	2023	Lian X. et al. (65)	86640 adult individuals, European	DN, 3,283 ncase 181,704 controls,European	129	OR=1.01(1.00-1.02)	P=0.5
Appendicular lean mass	2023	Ren L. et al. (66)	244730 adult individuals, European	DN, 3,283 ncase 181,704 controls,European	424	OR= 0.863(0.767-0.971)	P = 0.014
Grip strength left	2023	Ren L. et al. (66)	461026 adult individuals, European	DN, 3,283 ncase 181,704 controls,European	147	OR=1.119(0.688-1.820)	P=0.650
Grip strength right	2023	Ren L. et al. (66)	461089 adult individuals, European	DN, 3,283 ncase 181,704 controls,European	164	OR=0.847(0.552-1.300)	P= 0.447
Walking speed	2023	Ren L. et al. (66)	459915 adult individuals, European	DN, 3,283 ncase 181,705 controls,European	56	OR=0.495(0.206-1.189)	P=0.116
Periodontitis	2024	Yan P. et al. (67)	461031 adult individuals, European	DN, 3,283 ncase 210463 controls,European	6	OR=1.02(0.91–1.14)	P=0.77
coffee intake	2021	Mazidi M. et al. (74)	91462 adult individuals, European	DM eGFR,n = 133,413 individuals with replication in up to 42,166 individuals	5	beta=-0.00645	P=0.478
coffee consumption	2023	Fang J. et al. (75)	428860 adult individuals, European	DN, 3,283 ncase 210463 controls,European	33	OR:1.939 (1.012-3.712)	P =0.045
coffee consumption	2023	Fang J. et al. (75)	428860 adult individuals, European	T2DM with renal complications, 1,296 cases and 183,185 European-descent controls	35	OR=2.787 (0.926-8.394)	P = 0.047
coffee consumption	2023	Fang J. et al. (75)	428860 adult individuals, European	T1DM diabetes with renal complications, 963 cases and 183,185 controls	36	OR = 2.667 (0.796-8.929)	P = 0.112
coffee consumption	2023	Fang J. et al. (75)	428860 adult individuals, European	Urinary albumin-to-creatinine ratio in diabetes, 5,825 cases and 46061 controls of European individuals	30	OR=0.884 (0.395-1.802)	P=0.661
Sleep duration	2021	Mazidi M. et al. (81)	446118 adult individuals, European	eGFR in the total population, n = 133,413 individuals with replication in up to 42,166 individuals	78	beta=-0.019	p = 0.047
CTSC	2024	Zhou D. et al. (42)	31684 adult individuals, European	Diabetic nephropathy 1,032 case 451,248 control	12	OR=0.861	P=0.041
PDE5A	2024	Zhou D. et al. (42)	19173 adult individuals, European	Diabetic nephropathy 1,032 case 451,248 control	9	OR=0.842	P=0.018
inhibition of HMGCR	2024	Zhao R. et al. (48)	NOT REPORT	DN: males and females with 3283 cases and 181,704 controls	19	OR= 1.79	P = 0.01
COL6A2	2024	Zhang W. et al. (50)	49,708 individuals of Icelandic descent	DN:287,132 Finnish adult participants (3,676 cases and 283,456 controls)	23	OR=1.588 (1.284-1.963)	P=0.0000198
CBLN1	2024	Zhang W. et al. (50)	49,708 individuals of Icelandic descent	DN:287,132 Finnish adult participants (3,676 cases and 283,456 controls)	162	OR=1.141 (1.039-1.253)	P=0.00571
TGFBI	2024	Zhang W. et al. (50)	49,708 individuals of Icelandic descent	DN:287,132 Finnish adult participants (3,676 cases and 283,456 controls)	75	OR=1.284 (1.118-1.475)	P=0.000402
ITIH3	2024	Zhang W. et al. (50)	49,708 individuals of Icelandic descent	DN:287,132 Finnish adult participants (3,676 cases and 283,456 controls)	164	OR=1.179 (1.089-1.277)	P=0.0000506
ANG	2024	Gurung RL. et al. (51)	1,000 adult individuals,European+338 participants with Arab and Asian ethnicities.	YT2D DN(:≤40YAsian participants) Control=546, Case=321	1	OR=4.03 (1.28-12.68)	P = 0.017

DN, diabetic nephropathy; DKD, diabetic kidney disease; ESDR, End stage renal disease; YT2D, young onset of type 2 diabetes; eGFR, estimated glomerular filtration rate.
